# Assessing Individual- and Community-Level Variability in Predictors of Neonatal, Infant, and Under-Five Child Mortality in Ethiopia Using a Multilevel Modeling Approach

**DOI:** 10.3390/children9071071

**Published:** 2022-07-18

**Authors:** Kenenisa Abdisa Kuse, Teshita Uke Chikako, John Elvis Hagan, Abdul-Aziz Seidu, Bright Opoku Ahinkorah

**Affiliations:** 1 Department of Statistics, Bule Hora University, Bule Hora P.O. Box 144, Ethiopia; abdisakenenisa40@gmail.com; 2 Wondo Genet College of Forestry and Natural Resource, Hawassa University, Hawassa P.O. Box 05, Ethiopia; teshitauke@hu.edu.et; 3 Department of Health, Physical Education, and Recreation, University of Cape Coast, Cape Coast PMB TF0494, Ghana; 4 Neurocognition and Action-Biomechanics-Research Group, Faculty of Psychology and Sport Sciences, Bielefeld University, P.O. Box 10 01 31, 33501 Bielefeld, Germany; 5 College of Public Health, Medical and Veterinary Sciences, James Cook University, Townsville, QLD 4811, Australia; abdul-aziz.seidu@stu.ucc.edu.gh; 6 Centre for Gender, Takoradi Technical University, Takoradi P.O. Box 256, Ghana; 7 School of Public Health, Faculty of Health, University of Technology Sydney, Sydney, NSW 2007, Australia; bright.o.ahinkorah@student.uts.edu.au

**Keywords:** child mortality, community, EMDHS, Ethiopia, multivariable multilevel

## Abstract

**Background:** In low-and middle-income countries, child mortality rates are basic indicators of a country’s socio-economic situation and quality of life. The Ethiopian government is currently working to reduce child mortality to accomplish its long-term development goals. Using data from the Ethiopian Mini Demographic and Health Survey, 2019, this study analyzed the determinants of child mortality in Ethiopia. **Methods:** A total of 4806 children were considered in the final analyses. Multivariate analysis was used to estimate the effects of the predictors simultaneously on each child mortality outcome. **Results:** The findings revealed that 31.6% of children died during the neonatal stage, 39.1% during the infant stage, and 48.5% during the under-five stage. Variation in child mortality was discovered between Ethiopian community clusters, with the result of heterogeneity between clusters on newborn mortality (χ^2^ = 202.4, *p*-value < 0.0001), (χ^2^ = 777.35, *p*-value < 0.0001), and (χ^2^ = 112.92, *p*-value < 0.0001). Children’s neonatal, infant, and under-five mortality intracluster correlation coefficient (ICC) were 0.35, 0.33, and 0.36, respectively, across communities. **Conclusions:** In Ethiopia, under-five mortality remains a serious public health issue, with wide variations and high rates among community clusters. Intervention measures focusing on lowering rates of household poverty, increasing education opportunities, and improving access to health care could assist in reducing child mortality in Ethiopia.

## 1. Introduction

Child mortality rates are basic indicators of a country’s socio-economic and demographic situation and quality of life [1]. Global statistics show that approximately 2.4 million children died in the first month of life in 2020—approximately 6500 neonatal deaths every day—with about one-third of all neonatal deaths occurring within the first day after birth and close to three-quarters occurring within the first week of life [2]. In 2020, 5.0 million children under five years old died. This translates to 13,800 under-five children dying every day in 2020 [2].

In 2015, 5.9 million children under the age of five died worldwide. The World Health Organization (WHO) African countries still have the highest mortality rate of children under five years (81 per 1000 live births), about seven times the rate in the WHO European region (11 per 1000 live births). Low-income countries reported 76 deaths per 1000 live births, about 11 times the average rate in high-income countries (7 deaths per 1000 live births) [3]. The region with the highest under-five child mortality rate in the world is sub-Saharan Africa [4].

Within sub-Saharan Africa, Ethiopia has one of the highest rates of child mortality and morbidity in the world, with over 704 children dying every day from preventable illnesses [5,6]. With current trends, more than 3,084,000 children are likely to die by 2030 if sustainable measures are not taken. Specifically, the child mortality rate stands at 20 deaths per 1000 children surviving to age 12 months. The under-five mortality rate stands at 67 deaths per 1000 live births, and the neonatal mortality rate is 29 deaths per 1000 live births [7]. Additionally, there are regional differences in child mortality across the country, with prevalence rates ranging from 39 per 1000 live births in Addis Ababa to 125 per 1000 live births in Afar [3,4,5,7,8]. Although the 2016 Ethiopian Demographic and Health Survey report shows that childhood mortality rates have declined over time, compared to the reported under-five mortality rate of 116 deaths per 1000 live births 10–14 years prior to the survey (2002–2006), the rate of 67 deaths per 1000 live births in the 0–4 years prior to the survey (2012–2016) remains high [9].

The infant mortality rate in the fifty years before the survey was 43 deaths per 1000 live births. Child mortality was 12 deaths per 1000 live births to 12 months, while the overall under-five mortality rate was 55 deaths per 1000 live births. The neonatal mortality rate was 30 per 1000 live births [10,11].

Ethiopia’s situation still calls for concerted efforts by stakeholders, including scholars, to continuously generate updated empirical data to help evaluate existing healthcare systems and also to design appropriate sustainable interventions. To provide effective evidence-based interventions such as minimizing undue delays in accessing obstetric and neonatal health care services (e.g., enhancing community knowledge of neonatal death risk factors, reinforcing appropriate referral systems toward birth and complication-readiness strategy, decreasing transportation barriers, facilitating health-promoting behaviors [12]), the key role of updated scholarly evidence cannot be underestimated. Therefore, methodologically sound and nationally representative empirical studies are warranted to provide updated information to guide public policy. Therefore, this study used the 2019 Ethiopian Mini Demographic and Health Survey (EMDHS) data to assess the determinants of child mortality in Ethiopia.

## 2. Methodology

### 2.1. Data Source

The study used cross-sectional secondary data, which were obtained from the 2019 EMDHS. The survey drew a representative sample of children from the EDHS database at https://dhsprogram.com/data/available-datasets.cfm (accessed on 30 March 2022). The survey was conducted from 21 March 2019 to 28 June 2019 [10]. A total of 4806 children were considered in the final analyses.

### 2.2. Study Variables

#### 2.2.1. Outcome Variable

The outcome variable for this study was neonatal mortality (death in the first month of birth), infant mortality (death between birth and the first birthday), and under-five Mortality (death between birth and the fifth birthday), as mortality can occur because of age-specific reasons. Each of the three outcome variables were categorized into binary variables, which are split into “No death” or “Death”.

#### 2.2.2. Explanatory Variables

##### Individual-Level Factors

Individual level factors included size of a child at birth (very large, larger than average, average, smaller than average, very small); sex of child (male, female); mother’s educational level (no education, primary, secondary, higher); age of mother (15–24, 25–34, 35–44, 45 and above); father’s educational level (no education, primary, secondary, higher); mother’s occupational status (no, yes); household wealth index of (poorest, poorer, middle, richer, richest); sex of household head (male, female); current marital status (never in a union, married/living with partner, separated); breastfeeding duration (never breast fed, still breastfeeding, and ever breast fed); preceding birth interval (in months) (<24, ≥24); and birth order number (first, 2–4, >4). These explanatory variables were chosen based on previous literature, which found them to be associated with neonatal, infant, and under-five child mortality, and the report of 2019, EMDHS [6,7,11,12,13].

##### Community-Level Factors

Community-level factors included region (Tigray, Affar, Amhara, Oromia, Somalia, Benishangul-gumuz, SNNPR, Gambela, Harari, Dire Dawa, Addis Ababa) and place of residence (urban, rural).

### 2.3. Data Analysis Procedure

First, descriptive and bivariate analyses were performed. Using bivariate analysis, we selected candidate variables with *p*-values less than 0.25 for a multivariate multilevel logistic regression analysis [14,15].

#### 2.3.1. Multivariable Multilevel Logistic Analysis

Multivariable response multilevel models may possibly be necessary when one is interested in two or more outcomes measured on an individual’s within-group factors [16]. This study evaluated the effect of the predictors on the three outcomes of infant mortality and a single test of the combined effect of predictors.

#### 2.3.2. Multilevel Logistic Regression

##### Two Level Multilevel Analysis

Two-level multilevel logistic regression analysis was applied to assess the effects of individual and community level factors, for which individual factors were the first level (level 1), and community was the second level (level 2). The two-level logistic regression models were written as:

$$\log\left(\frac{\pi }{1-\pi }\right)=\beta_{0}+\beta_{1}X_{P}+U_{oj}$$

where π stands for the probability that the *i*th individual level factors in the *j*th community; $\beta_{0},\;\beta_{1},\;\beta_{2},\ldots ,\;\beta_{p}$ were the regression parameter of the variables associated with child mortality; $X_{1},\;X_{2,\ldots ,}X_{p}$ were independent variables related with each outcome variable and $U_{oj}$ (random effect at level 2).

Equivalently, we split model (1) into two: one for level 1 and the other for level 2.

$$\log\left(\frac{\pi }{1-\pi }\right)=\beta_{oj}+\beta_{1}X_{P}\;\;\mathrm{Model}:\;\mathrm{Level}\;\mathrm{One}$$

$$\beta_{oj}=\beta_{o}+U_{oj}\;\;\mathrm{Model}:\;\mathrm{Level}\;\mathrm{Two}$$

##### Multilevel Logistic Regression Empty Model

Multilevel empty model is a model that contains no explanatory variables at all that serves as a point of reference with which other models are compared. The two-level empty multilevel logistic regression model is expressed as follows:

$$\log it\left(\pi \right)=\beta_{0}+U_{oj}$$

where π is the probability (average proportion of successes) in group *j*; $\beta_{0}$ is the population average of the transformed probabilities; and $U_{oj}$ is the random deviation from this average for group *j*, and $U_{oj}\sim N\left(0,\;\sigma_{u}^{2}\right)$.

##### Random Intercept Multilevel Logistic Regression Model

With the random intercept model, the intercept is the only random effect, meaning that the groups differ in relation to the average value of child mortality, but the relationship between related factors and response variables cannot differ between them. The random intercept model expresses the log-odds, i.e., the logit of π as a sum of a linear function of the explanatory variables. That is,

$$\log\left(\frac{\pi }{1-\pi }\right)=\beta_{0j}+\beta_{1}X_{i1}+\beta_{2}X_{i2}\;+\;,\ldots ,\beta_{P}X_{iP}$$

where is π is the probability of child mortality, and the intercept term $\beta_{0}$ is assumed to vary randomly and is given by the sum of intercept $\beta_{0}$ and group-dependent deviations $U_{uj}$. That is, $\beta_{0j}=\beta_{0}+U_{oj}$.

##### Multi-Level Random Coefficient Logistic Regression Model

In this model, the coefficients of the explanatory variables are considered as random.

$$\log\left(\frac{\pi }{1-\pi }\right)=\beta_{o}+\beta_{1j}X_{i1}+\beta_{2j}X_{i2}\;+\;,\ldots ,\beta_{Pj}X_{iPj}+U_{oj}$$

Letting $\beta_{oj}=\beta_{o}+U_{oj},$ and $\beta_{hj}=\beta_{h}+U_{hj}$, *h* = 1,2,…, *k*, we can obtain

$$\log\left(\frac{\pi }{1-\pi }\right)=\beta_{o}\sum_{h=1}^{k}\beta_{h}x_{hij}+U_{oj}+\sum_{h=1}^{k}U_{hj}x_{hij}$$

$\beta_{o}\sum_{h=1}^{k}\beta_{h}x_{hij}$ is the fixed part of the model; $U_{oj}+\sum_{h=1}^{k}U_{hj}x_{hij}$ is the random part of the model where π is the probability of child mortality, and the intercept term $\beta_{0}$ is assumed to vary randomly and is given by the sum of intercept $\beta_{0}$ and group-dependent deviations $U_{uj}$. That is, $\beta_{0j}=\beta_{0}+U_{oj}$.

##### Multilevel Logistic Regression Parameter Estimation

The most common methods of estimating multilevel models used in this study are based on the likelihood approximations. Among the methods, marginal quasi-likelihood and penalized quasi-likelihood are the two prevailing approximation procedures. After applying these quasi-likelihood methods, the model is then estimated using iterative generalized least squares or reweighted iterative least squares consisting of Laplacian approximation, which is a full maximum likelihood estimation procedure in estimating random intercept and fixed effect [17].

Intra-class correlation coefficient (*ICC*): The authors purposively used *ICC* to measure the reliability of ratings for the clusters. Level one residuals distributed using the default logistic distribution $e_{ij}$ imply a variance of $\frac{\pi^{2}}{3}=3.29$. Thus, the variation of child mortality across community was determined. The *ICC* for the two-level binary data can be defined for each level separately:

$$ICC\left(\mathrm{Community}\right)=\frac{\delta^{2}{}_{\mathrm{Community}}}{\delta^{2}{}_{\mathrm{Community}}+\frac{\pi^{2}}{3}}$$

where $\frac{\pi^{2}}{3}$ denotes the variation of a lower-level unit (individual); $\delta^{2}{}_{\mathrm{Community}}$ indicates the differences between the communities.

### 2.4. Model Selection Criteria

#### Akaike Information Criteria (AIC)

Before fitting the model, checking the adequacy or goodness of fit of the model is needed. To select the best model, *AIC* (Akaike information criterion) was used. The model with a small value of AIC is the optimal model, which means the model is close to the actual one, and the model has few parameters to be estimated [17].

*AIC* are defined as:

$$AIC=-2ln\left(likelihood\right)+2k$$

where *k* indicates the degrees of freedom calculated from a variance-covariance matrix of the parameters, and *N* indicates the number of observations used in the estimation or, more precisely, the number of independent terms in the likelihood.

## 3. Results

### 3.1. Summary of Child Mortality Statistics in Ethiopia (Neonatal Mortality, Infant Mortality, and Under-Five Mortality)

Table 1 presents the descriptive results obtained from EDHS 2019 based on child mortality factors in Ethiopia, such as neonatal mortality, infant mortality, and under-five mortality.

Of the total 1520 children who died in the first month of birth, 189 resided in urban areas, and 1331 resided in rural areas. Out of the 1877 infant deaths, 377 occurred in urban areas, and 1500 in rural areas. Out of the 2337 under-five deaths, 361 occurred in urban areas, and 1970 in rural areas. Likewise, regarding deaths in the first month after birth, 770 children who died were males, and 750 females, respectively. From between birth and the first day, 938 males and 939 females died, respectively. For under-five deaths, 1195 were among males and 1136 females.

### 3.2. Inferential Statistical Analysis

#### 3.2.1. Bivariate Analysis

Before performing the multivariable multilevel analysis, a bivariate analysis was performed for all independent variables separately. Then, those variables showing association with child mortality at *p*-value < 0.25 were selected and entered into the multivariable multilevel analysis. Bivariate analysis in Table 1 shows that place of residence, mother’s educational level, sex of household head, wealth index of mothers, age of mothers, father’s educational level, region, birth order number, preceding birth interval (in months), mother’s occupational status, breastfeeding duration, and size of child at birth are significant factors for child mortality. However, current marital status was significant only with infant mortality but not neonatal and under-five mortality.

#### 3.2.2. Multivariable Multilevel Logistic Analysis

##### Multilevel Analysis using Empty Model

Before performing the multilevel analysis, there was a test of heterogeneity performed on each child mortality outcome separately. The parametric version of assessing the heterogeneity of child mortality was utilized.

From Table 2, the variances of neonatal, infant, and under-five mortality were estimated as $\sigma_{ou}{}^{2}$ = 2.283, $\sigma_{ou}{}^{2}=2.905,$ and $\sigma_{ou}{}^{2}=0.186$, respectively, which were significant at 5% level of significance and indicate that there were variations of child mortality across the community of Ethiopia. Accordingly, the intra-cluster correlation coefficients (ICCs) from the empty model were 0.409, 0.468, and 0.053 for neonatal, infant, and under-five mortality across the community, respectively. This shows that about 40.9%, 46.8%, and 5.3% of the variation in neonatal, infant, and under-five mortality can be attributed to community-level variations, respectively.

### 3.3. Model Comparison

Once the set of candidate models were chosen, the statistical analysis selected the best fit of these models. A good model selection technique was to balance goodness of fit with simplicity. Therefore, choosing a relevant multilevel model is an important step, and it should be based on the necessity of parsimony in the model.

Table 3 shows that AIC for neonatal, infant, and under-five mortality was small for model III (individual- and community-level factors). This shows that model III (individual- and community-level factors) best-fitted the data. Therefore, model III (individual- and community-level factors) was used for further analysis and interpretation.

Mother’s educational level, wealth index of mothers, father’s educational level, preceding birth interval, duration of breastfeeding, region, place of residence, and sex of household head has a significant impact on child mortality at a 5% level of significance. The intra-cluster correlation (ICC) of neonatal mortality, infant mortality, and under-five mortality across the community was 0.35, 0.33, and 0.36, respectively (Table 4). These observed correlations indicate that about 35% of the variation in neonatal mortality is due to the variation across the community, about 33% of the variation in infant mortality is due to the variation across the community, and about 36% of the variation in under-five mortality is due to the variation across the community.

### 3.4. Neonatal Mortality

The results in Table 4 show the neonatal mortality for mothers with secondary and higher educational levels was about 22% and 46% less likely compared to mothers with no education, respectively. Similarly, the probability of neonatal death for children from the poorer, middle, richer, and richest households was about 82%, 93%, 88%, and 26% less likely compared to children from the poorest families, respectively. Regarding fathers’ educational levels, a child from a father with a primary, secondary, and higher educational level was about 66%, 25%, and 59% less likely to die within the first month of life compared to a child from a father who has no education, respectively. The odds of neonatal mortality for the children from Afar, Somali region, Harari, Addis Ababa, and Dire Dawa were about 26%, 77%, 30%, 68%, and 33% less likely compared to children from the Tigray region, respectively, while a child from the Amhara, Oromia, Benishangul, SNNP and Gambela regions was 4.89, 8.71, 10.28, 1.94, and 1.46 times more likely to succumb to neonatal mortality compared to a children from the Tigray region, respectively. Regarding area of residence, a child from a rural area of the country was about 1.05 more likely to die at the neonatal stage compared to a child from the urban area of the country. Similarly, the odds of the neonatal mortality were decreased by 41% for a child with a female household head.

### 3.5. Infant Mortality

The odds of infant mortality were decreased by 64%, 88%, and 82%, respectively, for the mothers with primary, secondary, and higher educational levels. Children from the richer and richest families were 63% and 55% less likely to die in infancy compared with children from the poorest families, respectively. Children whose fathers have primary education were 1.12 times more likely to die in infancy compared to a child whose father has no education. Children with preceding birth interval greater or equal to 24 months are 46% less likely to die in infancy compared to children with preceding birth interval less than 24 months. The odds of infant mortality for children from Addis Ababa were 54% less compared to children from the Tigray region, while a child from Somali, Benishangul, SNNP, and Gambela regions was 3.26, 5.45, 0.74, and 21.2 times more likely to succumb to infant mortality compared to a child from the Tigray region, respectively. The odds of infant mortality were increased 1.28 times for children from rural areas of the country. Similarly, the infant mortality rate for children of female householders decreased by 19%.

### 3.6. Under-Five Mortality

The odds of under-five mortality were decreased by 25% for children from mothers with a secondary educational level. Children from the richer and richest families were 27% and 73% less likely to die at under five years compared with children from the poorest families, respectively. Children whose fathers have higher education were 28% less likely to die at under five years compared to children whose fathers have no education. Regarding preceding birth interval, children with preceding birth interval greater or equal to 24 months were 16% less likely to die at under five years compared to children with preceding birth interval less than 24 months. The odds of under-five mortality for the children from Oromia, Somali, Gambela, and Addis Ababa were 14%, 27%, 45%, and 43% less compared to children from the Tigray region, respectively. The odds of under-five mortality were decreased by 46% for children from rural areas of the country. Similarly, the odds that a female householder’s child will die before the age of five was reduced by 67%.

## 4. Discussion

This study used a multivariable multilevel logistic regression model to determine the effect of each independent variable on the child mortality outcome variable. Thus, this study aimed at examining the major influential factors on child mortality, such as mothers’ educational level, wealth index of mothers, fathers’ educational level, preceding birth interval, duration of breastfeeding, region, place of residence, and sex of household head.

Mother’s educational level significantly influenced child neonatal, infant, and under-five mortality. Children with mothers who have no formal education or are illiterate were more likely to die than those with mothers who have a secondary educational level. Similar findings conducted in Calverton [18] suggest that children of mothers who have primary education levels are less likely to die than children of mothers with no education or who are illiterate, and children of mothers with secondary and higher education levels were less likely to die. A similar argument suggested by Griffiths et al. suggests that more-educated mothers can better utilize their limited resources than mothers with little or no education and have better health and parenting practices, which lead to better child survival and may have better information on health. [19].

It was also found that preceding birth interval was significant to child neonatal, infant, and under-five mortality. This outcome means short preceding birth intervals and high parity largely increase child neonatal, infant, and under-five mortality risk after accounting for unobserved heterogeneity between them [20]. Previous research conducted in sub-Saharan Africa countries suggests that this observation could be related to maternal depletion syndrome and resource competition between the siblings in addition to lack of care and attention experienced by high-order children [21,22,23].

The sex of the household head was another significant factor in this study. Children from female-headed households were less likely to die than those from male-headed households regarding the child neonates, infants, and those under five years. Similar findings from Nepal and Nigeria [24,25] show that women from female-headed households were less likely to experience the death of a child than women from male-headed households. Hence, women could talk more easily with the female heads of the household, especially about their reproductive health problems and children’s illnesses. The similar study conducted in India [26] reiterates that more female autonomy results in a significant reduction in child mortality because of the greater prenatal care and greater possibility of delivery in the hospital.

The father’s educational level was significant. It is logical to infer that educated fathers usually improve the agency to influence family and child-care decisions. Previous studies conducted in Bangladesh and Comoros by [27,28,29] contradict with this identified association and argued that an educated father is likely to have a better income, which plays a key role in the financial strength of the family and the provision of necessary health services for the family. Similarly, in this study, the factor of wealth index of mothers was significant. This result is consistence with other studies carried out by Gizachew, Addisalem et al. and Desalegn et al. [30,31,32]; however, a study conducted in Ethiopia [33] contradicts this finding and suggested that the wealth index of the household is not significant to child mortality. Wealthier families have better living standards or quality of life and better accessibility of health services. This status helps to reduce the likelihood of child mortality among wealthier families [34]. Comparatively, households with lower wealth status are less likely to have availability of health services and access to basic facilities of sanitation, hygienic toilets, and clean drinking water [35,36].

For place of residence, child mortality of families who resided in rural areas was higher than those who resided in urban areas. Usually, health infrastructural efforts nad basic social amenities made in rural areas are not complemented by work done in urban areas. Similar findings from Tsala et al. and Honwana and Melesse [37,38] suggest that living in urban areas decreased the risk for child mortality as compared to living in rural areas because of specific individual level or socio-ecological factors. Living in urban areas serves as a further protective factor for mothers whose children previously died and were more educated, and also reduced the promoting effects of older ages in mothers [39,40]. Even so, living in urban areas also reduced the health-promoting alternative of the poor and good-structural quality of housing. In addition, permanent housing quality in urban areas offered a risk factor for neonates, infants, and under-five child mortality as compared to housing conditions in rural areas [41].

### Strengths and Limitation

This study shares some of the limitations inherent in the use of cross-sectional datasets. Since respondents were interviewed at a point in time, the opportunity to establish a cause–effect relationship between predictor and outcome variables (neonates, infants, and under-five child mortality) is lost. More so, through the analysis in this study, more vigorous and thorough information would have been provided if there had been variables measuring other child indicators. However, throughout this study, the datasets considered were limited to the indicators discussed. Despite these limitations, the study analyzed child mortality problems through the three mortality outcomes and provided valuable information on the factors that are associated with child mortality in Ethiopia.

## 5. Conclusions

The problem of neonatal, infant, and under-five mortality is a serious problem in Ethiopia. The results revealed that factors influencing this phenomenon include mother’s educational level, wealth index of mothers, father’s educational level, preceding birth interval, duration of breastfeeding, region, place of residence, and sex of household head. Stakeholders should aim at reducing child mortality in the country by incorporating these factors through appropriate intervention programmes (e.g., health education and promotion, provision of basic social amenities, especially in rural areas). Such interventions, including policies should target these characteristics to improve child health outcomes in Ethiopia.

## Figures and Tables

**Table 1:** Results of descriptive statistics and bivariate analysis.

Variables	Categories	Child Mortality								
		Neonatal Mortality			Infant Mortality			Under-Five Mortality		
					Death	Alive	$\chi^{2}$	Death	Alive	$\chi^{2}$
		Death	Alive	$\chi^{2}$						
Place of residence	Urban	189	675	46.327 *	377	487	9.279 *	361	503	19.04 *
	Rural	1331	2611		1500	2442		1970	1972	
Mothers’ educational level	No education	1022	1984	52.273 *	1131	1875	6.157 *	1544	1462	39.37 *
	Primary	394	859		530	723		583	670	
	Secondary	79	281		134	226		133	227	
	Higher	25	162		82	105		71	116	
Sex of household head	Male	1253	2594	7.940 *	1536	2311	6.157 *	1866	1981	12.5 *
	Female	267	692		341	618		465	494	
Wealth index of mothers	Poorest	1574	1135	56.553 *	637	1072	10.040 *	847	862	67.305 *
	Poorer	259	531		306	484		454	336	
	Middle	242	432		268	406		355	319	
	Richer	231	423		244	410		287	367	
	Richest	214	765		422	557		388	591	
Age of mothers	15–24	781	1584	11.646 *	925	1440	7.264 *	1181	1184	5.790 *
	25–34	676	1495		828	1343		1012	1159	
	35–44	59	186		115	130		125	120	
	45 and above	4	21		9	16		13	12	
Current marital status	Never in Union	2	6	0.640	3	5	0.064	5	3	0.712
	Married	1470	3183		1816	2837		2254	2399	
	Separated	48			58	87		72	73	
Fathers’ educational level	No education	792	1497	41.640 *	848	1441	10.177 *	1206	1083	50.709 *
	Primary	505	1060		652	913		750	815	
	Secondary	130	378		211	297		213	295	
	Higher	93	351		166	278		162	282	
Region	Tigray	171	326	62.496 *	213	284	21.498 *	251	246	41.387 *
	Afar	159	322		164	317		238	243	
	Amhara	170	311		181	300		266	215	
	Oromia	187	377		225	339		273	291	
	Somalia	231	469		275	425		341	359	
	Benishangul-gumuz	106	294		139	261		214	186	
	SNNPR	266	446		297	415		348	364	
	Gambela	84	232		121	195		113	203	
	Harari	58	157		101	114		89	126	
	Addis Ababa	34	200		81	153		102	132	
	Dire Dawa	54	152		80	126		96	110	
Mothers’ working status	Not working	1136	2313	9.693 *	1328	2313	11.561 *	1167	1772	12.72 *
	Working	384	973		549	973		654	703	
Birth order number	1(First)	242	642	13.490 *	347	642	10.994 *	412	472	5.874 *
	2–4	693	1527		881	1527		1054	1166	
	>4	585	1117		649	1117		865	837	
Sex of child	Male	770	1647	0.120	938	1479	0.125	1195	1222	1.719
	Female	750	1639		939	1450		1136	1253	
Preceding birth interval (months)	<24	378	857	8.799 *	471	764	9.588 *	648	587	10.476 *
	≥24	1142	2429		1406	2165		1683	1888	
Duration of breastfeeding	Ever breastfed, not currently breastfeeding	785	1732	13.148 *	988	1529	12.097 *	1322	1195	41.733 *
	Never breastfed	48	132		61	119		97	83	
	Still breastfeeding	687	1422		828	1281		912	1197	
Size of child at birth	Very large	253	543	8.401 *	332	464	9.587 *	358	438	11.22 *
	Larger than average	226	460		277	409		334	352	
	Average	638	1409		781	1266		974	1073	
	Smaller than average	139	334		174	299		245	228	
	Very small	264	540		313	491		420	384	

* Chi-square was significant at (*p* < 0.25).

**Table 2:** Results of multivariate multilevel logistic regression of empty model.

Response Variable	Indicators	Estimate	S.E	Z-Value	*p*-Value	95% CI
Neonatal Mortality	$\beta_{O}$ = Intercept	5.418	2.488	3.68	0.000	2.202–13.327
	Community	Estimate				
	Var($U_{oj})$ = $\delta_{O}{}^{2}$	2.283	0.997			0.970–5.374
	ICC for Community	0.409	0.105			0.227–0.620
	Deviance-based chi-square $(X^{2})=$ 927.50; *p*-value < 0.0001					
Infant Mortality	$\beta_{O}$ = Intercept	24.093	12.65	6.06	0.000	8.604–67.464
	Community	Estimate				
	Var($U_{oj})$ = $\delta_{O}{}^{2}$	2.905	1.294			1.213–6.957
	ICC for Community	0.468	0.110			0.269–0.678
	Deviance-based chi-square $(X^{2})=$ 777.35; *p*-value < 0.0001					
Under-five Mortality	$\beta_{O}$ = Intercept	0.499	0.067	−5.15	0.000	0.383–0.650
	Community	Estimate				
	Var($U_{oj})$ = $\delta_{O}{}^{2}$	0.186	0.087			0.073–0.469
	ICC for Community	0.053	0.023			0.021–0.124
	Deviance-based chi-square $(X^{2})=$ 112.92; *p*-value < 0.0001					

**Table 3:** Model comparison between the models on child mortality.

Response	Model Comparison Criteria	Null Model (Model I)	Individual-Level Factors (Model II)	Individual- and Community-Level Factors (Model III)
Neonatal mortality	AIC	3552.244	2912.324	2633.845
Infant Mortality	AIC	1605.123	1574.248	1359.224
Under-five Mortality	AIC	6114.218	5999.25	5963.110

**Table 4:** Results of Multivariable Multilevel Logistic Regression of Random coefficient.

	Neonatal Mortality		Infant Mortality		Under-five Mortality	
Fixed Part	AOR (95% CI)	*p*-Value	AOR (95% CI)	*p*-Value	AOR (95% CI)	*p*-Value
Constant	0.47 [0.31–0.71]	0.000 *	1.44 [1.44–7.06]	0.000 *	4.51 [0.92–22.0]	0.062
**Mothers’ education level** (ref. no education)						
Primary	0.89 [0.67–1.64]	0.658	0.36 [0.24–0.68]	0.001 *	0.97 [0.82–1.13]	0.715
Secondary	0.78 [0.57–0.96]	0.042 *	0.12 [0.04–0.23]	0.000 *	0.75 [0.54–0.88]	0.002 *
Higher	0.54 [0.32–0.89]	0.000 *	0.18 [0.09–0.26]	0.000 *	0.84 [0.56–1.27]	0.427
**Wealth index of household** (ref. poorest)						
Poorer	0.18 [0.12–0.27]	0.000 *	0.96 [0.79–2.34]	0.227	1.05 [0.86–1.29]	0.578
Middle	0.07 [0.03–0.16]	0.000 *	0.97 [0.97–1.20]	0.609	0.81 [0.57–1.24]	0.403
Richer	0.12 [0.09–0.19]	0.000 *	0.37 [0.18–0.66]	0.004 *	0.27 [0.05–0.54]	0.011 *
Richest	0.74 [0.55–0.99]	0.000 *	0.45 [0.09–0.52]	0.001 *	0.77 [0.77–0.83]	0.001 *
**Age of mothers** (ref. 15–24)						
25–34	0.98 [0.86–1.11]	0.118	0.96 [0.84–1.08]	0.968	0.90 [0.80–1.02]	0.128
34–44	0.86 [0.62–1.18]	0.096	0.75 [0.91–1.29]	0.884	1.23 [0.92–1.64]	0.145
40 and above	0.63 [0.21–1.93]	0.254	1.09 [0.47–2.54]	0.771	1.26 [0.55–2.88]	0.568
**Fathers’ educational level** (ref. no education)						
Primary	0.34 [0.26–0.84]	0.031 *	1.12 [1.02–2.56]	0.000 *	0.89 [0.77–1.03]	0.148
Secondary	0.75 [0.52–0.99]	0.000 *	1.08 [0.86–1.36]	0.156	0.82 [65–1.03]	0.092
Higher	0.41 [0.29–0.87]	0.000 *	0.81 [0.62–1.07]	0.092	0.72 [0.55–0.95]	0.022 *
**Mothers’ working status** (ref. no)						
Yes	0.90 [0.78–1.05]	0.142	1.04 [0.91–1.20]	0.365	1.04 [0.91–1.20]	0.509
**Birth Order Number** (ref. First)						
2–4	1.06 [0.88–1.27]	0.114	1.07 [0.90–1.26]	0.984	0.93 [0.79–1.10]	0.432
>4	1.08 [0.89–1.33]	0.069	1.58 [0.93–2.99]	0.465	0.99 [0.82–1.19]	0.933
**Preceding birth interval (in months)** (ref. <24 months)						
≥24 months	0.66 [0.54–0.96]	0.000 *	0.54 [0.32–0.84]	0.000 *	0.84 [0.72–0.97]	0.019 *
**Duration of breast feeding** (ref. ever breastfed, not currently breastfeeding)						
Never breast feeding	0.68 [0.43–0.99]	0.025 *	0.56 [0.32–0.74]	0.018 *	1.08 [0.79–1.47]	0.620
Still breast feeding	1.05 [0.93–1.20]	0.901	0.98 [0.87–1.11]	0.621	0.68 [0.60–0.77]	0.000 *
**Size of child at birth** (ref. very large)						
Larger than average	1.04 [0.83–1.30]	0.254	0.97 [0.79–1.20]	0.961	1.09 [0.88–1.34]	0.410
Average	0.95 [0.80–1.14]	0.982	0.88 [0.74–1.05]	0.721	1.05 [0.88–1.24]	0.562
Smaller than average	0.83 [0.64–1.07]	0.145	0.88 [0.69–1.12]	0.451	1.22 [0.97–1.55]	0.087
Very small	0.97 [0.78–1.21]	0.632	0.97 [0.97–1.19]	0.632	1.41 [0.93–1.76]	0.530
**Region**: (ref. Tigray)						
Afar	0.74 [0.52–0.92]	0.008 *	0.77 [0.58–1.02]	0.078	0.56 [0.37–1.42]	0.079
Amhara	4.89 [2.24–10.3]	0.000 *	0.86 [0.66–1.12]	0.279	1.10 [0.85–1.44]	0.438
Oromia	8.71 [5.55–9.21]	0.000 *	0.92 [0.71–1.18]	0.522	0.86 [0.58–0.91]	0.000 *
Somali	0.23 [0.09–0.17]	0.040 *	3.26 [2.59–11.4]	0.000 *	0.73 [0.56–0.94]	0.016 *
Benishangul-gumuz	10.64 [5.51–23.9]	0.000 *	5.45 [1.83–20.4]	0.000 *	1.02 [0.78–1.35]	0.841
SNNPR	1.94 [1.32–2.86]	0.009 *	0.74 [0.77–1.25]	0.000 *	0.92 [0.72–1.17]	0.504
Gambela	1.46 [1.26–3.77]	0.000 *	21.2 [9.30–121.2]	0.000 *	0.55 [0.41–0.76]	0.000 *
Harari	0.70 [0.49–0.96]	0.022 *	0.85 [0.85–1.16]	0.333	0.69 [0.57–1.13]	0.220
Addis Ababa	0.32 [0.21–0.48]	0.006 *	0.46 [0.32–0.67]	0.000 *	0.57 [0.21–0.82]	0.001 *
Dire Dawa	0.67 [0.47–0.97]	0.000 *	1.02 [0.73–1.43]	0.884	0.99 [0.68–1.43]	0.981
**Place of residence** (ref. urban)						
Rural	1.05 [1.01–2.44]	0.000 *	1.28 [1.05–3.29]	0.008 *	0.54 [0.36–0.92]	0.000 *
**Sex of household head** (ref. male)						
Female	0.59 [0.34–0.94]	0.042 *	0.81 [0.68–0.95]	0.012 *	0.33 [0.09–0.44]	0.000 *
**Random Part**						
Var (Cons.) community	1.772		1.628		1.884	
ICC for community	0.35		0.33		0.36	

* significant at (*p* < 0.05).

## Data Availability

The dataset used for the study is available at: https://dhsprogram.com/data/available-datasets.cfm (accessed on 30 March 2022).
